# Effectiveness of non-medical health worker-led counselling on psychological distress: a randomized controlled trial in rural Nepal

**DOI:** 10.1017/gmh.2019.15

**Published:** 2019-07-16

**Authors:** N. Markkula, V. Lehti, P. Adhikari, S. Peña, J. Heliste, E. Mikkonen, M. Rautanen, E. Salama, B. Guragain

**Affiliations:** 1 Department of Psychiatry, Helsinki University and Helsinki University Hospital, Helsinki, Finland; 2 Physicians for Social Responsibility – Finland, Helsinki, Finland; 3 Programa de Estudios Sociales en Salud, Facultad de Medicina Clínica Alemana Universidad del Desarrollo; 4 Research Centre for Child Psychiatry, University of Turku, Turku, Finland; 5 Department of Public Health Solutions, the National Institute for Health and Welfare, Helsinki, Finland; 6 Centre for Victims of Torture, Kathmandu, Nepal; 7 Facultad de Medicina, Universidad Diego Portales, Santiago, Chile; 8 Institute of Biomedicine, University of Turku, Turku, Finland; 9 Institute for Molecular Medicine Finland, University of Helsinki, Helsinki, Finland; 10 Psychiatric Hospital for Prisoners, Vantaa, Finland; 11 Department of Child Psychiatry, Turku University Hospital and University of Turku, Turku, Finland; 12 Doctoral Programme in Clinical Research, University of Turku, Turku, Finland

**Keywords:** Common mental disorders, lay counselling, Nepal, randomized controlled trial, task shifting

## Abstract

**Background.:**

An essential strategy to increase coverage of psychosocial treatments globally is task shifting to non-medical counsellors, but evidence on its effectiveness is still scarce. This study evaluates the effectiveness of lay psychosocial counselling among persons with psychological distress in a primary health care setting in rural Nepal.

**Methods.:**

A parallel randomized controlled trial in Dang, rural Nepal (NCT03544450). Persons aged 16 and older attending primary care and with a General Health Questionnaire (GHQ-12) score of 6 or more were randomized (1:1) to receive either non-medical psychosocial counselling (PSY) or enhanced usual care (EUC). PSY was provided by lay persons with a 6-month training and consisted of 5-weekly counselling sessions of 35–60 min with a culturally adapted solution-focused approach. EUC was provided by trained primary health workers. Participants were followed up at 1 (T1) and 6 months (T2). The primary outcome, response to treatment, was the reduction of minimum 50% in the Beck Depression Inventory (BDI) score.

**Results.:**

A total of 141 participants, predominantly socially disadvantaged women, were randomized to receive PSY and 146 to EUC. In the PSY, 123 participants and 134 in the EUC were analysed. In PSY, 101 participants (81.4%) had a response compared with 57 participants (42.5%) in EUC [percentage difference 39.4% (95% CI 28.4–50.4)]. The difference in BDI scores at T2 between PSY and EUC was −7.43 (95% CI −9.71 to −5.14).

**Conclusions.:**

Non-medical (lay) psychosocial counselling appears effective in reducing depressive symptoms, and its inclusion in mental health care should be considered in low-resource settings.

## Introduction

There is a vast treatment gap in mental disorders globally: approximately one in four persons with common mental disorders has received treatment (Alonso *et al*., 2018; Araya *et al*., 2018). The gap is wider in developing countries, where human resources for mental health are extremely scarce (Chisholm *et al*., 2016*b*; De Silva *et al*., 2014; Kakuma, 2011). In 2011 there was an estimated lack of 1.2 million mental health professionals in low and middle-income countries (Kakuma, 2011), and since then the global number of psychiatrists has decreased or remained stable (WHO, 2018).

Psychosocial interventions, also in their culturally adapted forms, have been shown to be effective in the treatment of common mental disorders (Chowdhary *et al*., 2014; De Silva *et al*., 2013). However, extending the availability of psychosocial interventions is particularly challenging, as they depend solely on the availability of human resources. Also, the focus on disease-specific treatment packages and stigma contribute to the low coverage of psychological therapies (Singla *et al*., 2017). Providing psychosocial counselling for common mental disorders, without disease-specific focus, at a primary care setting might help overcome some of these challenges.

One essential strategy to tackle this challenge of lacking workforce is task shifting (or task sharing), meaning ‘delegating tasks to existing or new cadres with either less training or narrowly tailored training’ (Kakuma, 2011; Shidhaye *et al*., 2015). Traditionally, lay (non-medical) health workers have occupied supporting roles in mental health care, providing support to family members, ensuring adherence and assisting in outreach activities (Kakuma, 2011). Nevertheless, there are promising results of their role in providing psychological treatment (van Ginneken *et al*., 2013). A systematic review reported 27 trials of psychological treatments provided by nonspecialist providers with a combined effect size of 0.49 in favour of the intervention (Singla *et al*., 2017). Other systematic reviews have also been carried out with similar findings (Joshi *et al*., 2014). An economic evaluation of one intervention showed it to be not only cost-effective but also cost-saving (Buttorff *et al*., 2012).

Some limitations of the existing evidence are its lack of scale-up measures, lack of indigenous elements and the fact that a majority of studies have focused exclusively on women (Singla *et al*., 2017). The requirement of ongoing, structured supervision provided by experts has been considered a particular challenge for scale-up, and peer supervision could be one potential solution. Exploration of which indigenous elements would be effective in lay counselling was considered a key element for further research (Singla *et al*., 2017). In this study in rural Nepal, we expanded previous research by using flexible, non-structured supervision and included indigenous elements such as breathing exercises and relaxation to strengthen the cultural pertinence of the intervention.

Nepal is a low-income country facing many economic, political, environmental and social challenges, and has a high burden of mental disorders (Luitel *et al*., 2013). The treatment gap is particularly wide: according to a general population study, only 8% of persons with current depression had received any treatment, and only 2% attended primary care, while others had sought help from traditional healers and mental health professionals (Luitel *et al*., 2017). The barriers to seeking treatment relate to stigma and the high cost of care. There is a vast lack of human resources in the mental health sector: there are approximately 50 psychiatrists and 20 psychologists in a country of 29 million population (Luitel *et al*., 2015). Primary care workers are perceived as overburdened with multiple duties, and there is a lack of psychotropic medications and of monitoring and supervision in mental health (Luitel *et al*., 2015).

The low available resources and high demand for mental health care highlight the need to assess alternative options for delivering effective treatments to psychological problems in Nepal. This study aimed to assess the effectiveness of psychosocial counselling as practised by non-medical psychosocial counsellors in improving the mental health outcomes of persons with psychological distress in a primary health care setting in rural Nepal.

## Methods

### Study design

We conducted a parallel randomized controlled trial with a 1:1 allocation ratio. The study has been registered in clinicaltrials.gov (NCT03544450) and no changes in the methods occurred after trial registration. The Consolidated Standards for Reporting Trials (CONSORT) were followed for reporting the trial.

### Setting

The study was carried out in Dang district, Western Nepal. Dang has a population of approximately 550 000 and was one of the most affected regions in the Nepal Maoist conflict (1996–2006). The intervention was carried out in two government health posts: Sisaniya and Gadawa health centres (Sisaniya has been upgraded to district hospital since the trial). These health posts were chosen because they were considered to represent typical characteristics of the region, and were willing to cooperate in the study and provide the necessary physical space and assistance. The health posts cover areas of 21 000 and 12 500 persons, respectively, and are staffed by health assistants, auxiliary nurse-midwives, nurses and midwives, and in Sisaniya also a medical doctor and administrative staff. The health workers have 1.5–3 years of formal training and are hired by the Ministry of Health.

The study was carried out as part of a development cooperation project named ‘Developing a Community Model of Mental Health Care in Nepal’ funded by the Ministry for Foreign Affairs, Finland. The project aimed to increase the availability of effective treatment for mental health problems at government health facilities in Dang through outreach activities in the community; empowerment and advocacy efforts; training primary health care workers in detection and treatment, and providing psychosocial counselling.

Ethical permission was granted by the Nepal Health Research Council (NHRC).

### Participants

Adults visiting Sisaniya and Gadawa health posts were informed about the study by the health workers and invited to participate. A written informed consent was obtained from potential participants, and consenting participants were then screened for eligibility by two research assistants. Inclusion criteria were: (1) age 16 years or older, (2) scoring 6 or above on the General Health Questionnaire (GHQ-12) (Koirala *et al*., 1999), (3) being able to fluently communicate in Nepali, (4) residence in Dang for the subsequent 10 months. The GHQ was scored as 0 or 1 for each question (GHQ scoring method), and a score of 7 or higher was defined as psychological distress (Patel *et al*., 2008). Persons with severe illnesses or conditions requiring urgent attention, such as psychotic symptoms or suicidality, were excluded and provided appropriate treatment (consultation of a psychiatrist and medication, if necessary). Suicidality was assessed with the question ‘Do you often think a lot about death, either your own, someone else's, or death in general?’, and history of a psychotic disorder with the question ‘Has the person previously experienced a psychotic episode?’. This question was usually presented to the accompanying person. Current psychotic symptoms were assessed clinically. Eligible participants were invited to take part in the RCT.

### Intervention

The intervention was psychosocial counselling [19] consisting of five 45-min appointments, two in the first week and weekly meetings in weeks 2–4. The intervention focuses on problem-solving, emotional support and coping strategies, and skills. On a theoretical level, the training programme was influenced by the principles of medical anthropology by Arthur Kleinman, such as being aware and encouraging traditional practices and local explanatory models and idioms of distress (Tol *et al*., 2005). The content of the training was tailored to individual participant needs, but included always the following components: (i) introduction, explanation, and rapport building; (ii) assessment of an understanding of the problem (including looking for positive assets); (iii) goal setting (asking the client what outcomes are preferred); (iv) problem management (exploring and identifying solutions, brainstorming, working with existing coping strategies, using social and cultural resources, and additional techniques such as relaxation and psycho-education); (v) implementation (making a plan of action and transition); and, finally, (vi) termination of counselling. Details on the intervention have been published elsewhere (Jordans *et al*., 2003; Tol *et al*., 2005).

The counsellors delivering the intervention were lay persons with a minimum of 12 years of education completed who had received a 6-month training in psychosocial counselling provided by Centre for Victims of Torture. The training included components of theoretical background, basic therapeutic skills, components of cognitive-behavioural therapy, problem-solving, exposure therapy, yoga and meditation. Other requirements for the counsellors were work experience in counselling (minimum 3 years), knowledge of Tharu language spoken in Dang, good communication skills, and ability to apply different therapeutic techniques. Additionally, persons already living in Dang were prioritised in the recruitment, to increase the sustainability of the intervention.

If needed, the counsellors had the opportunity to receive advice from senior counsellors or project district coordinator in person or a psychologist or medical doctor over the phone. Local and telephone supervision was carried out as needed, typically 1–3 times a week. Additionally, field supervision was organized every 2 months, when the team visited Dang. In the supervision meetings or phone calls, challenging cases were discussed, the supervisors helped the counsellors identify the main problem, and suggested suitable techniques to address the problem.

### Enhanced usual care

The comparator was enhanced usual care (EUC). EUC refers to the care available at the health posts given by health workers who had been trained by the development cooperation project run by Centre for Victims of Torture and Physicians for Social Responsibility – Finland in which this study took place. The health workers at both health posts had received a 5-day training and a 3-day refresher training in detection and treatment of mental disorders. Psychotropic medication is supposedly available at the health posts according to government guidelines, but in practice the supply is inconsistent. Participants in both arms were free to seek help at the health post.

### Outcomes

All participants were assessed at the beginning of the intervention (T0), immediately after the study (T1, 1 month) and after 6 months (T2) with the following instruments: (1) the Beck Depression Inventory (BDI), 21 item version (Beck *et al*., 1961; Kohrt *et al*., 2002), (2) the Beck Anxiety Inventory (BAI) (Beck *et al*., 1988; Kohrt *et al*., 2004); and (3) the WHO Disability Assessment Schedule (WHODAS) 12 item version 2.0 (Üstün *et al*., 2010).

The primary outcome, response to treatment, was defined as a reduction of minimum 50% in the BDI 21-item version (score range 0–63) from T0 to T2. The BDI is a self-reported depression questionnaire which been extensively validated. The study used the Nepali version, which showed acceptable overall validity and reliability (*α* 0.85) (Kohrt *et al*., 2002). Using a cut-off of 16/17, the specificity is 0.86 and sensitivity 0.85. Items related to appetite loss, libido and being punished were considered more problematic in terms of validity and reliability, but their removal was not recommended. Secondary outcomes were mean reductions in BDI, BAI, and WHODAS. The Nepali translation of the BAI has also shown good validity and similar reliability to Western contexts (*α* 0.89), even though items related to somatic symptoms appeared to reduce the internal validity somewhat (Kohrt *et al*., 2004). The WHODAS, even though it has not been validated in Nepal, has been used in other studies, and it was chosen for international comparability (Jordans *et al*., 2019).

The initial trial sample size calculation was made with the plan of using BDI as a continuous outcome variable. However, later the primary outcome was defined as a 50% reduction in the BDI since this outcome is widely used in other studies, has been validated as corresponding to a clinical assessment of response (Riedel *et al*., 2010). No changes in the primary outcome were made after data collection began.

Data were collected by two research assistants who interviewed the participants and filled in the data in paper format and then entered it into an electronic data sheet. The research assistants had received a 10-day training in research methodology and basic psychosocial support.

### Sample size

The sample size calculation aimed to detect a difference of three points on the BDI, leading to a minimum sample size of 132 participants in each group (power  = 0.80, significance level 5%, two-tailed test). Considering the possible 30% attrition, the target for recruitment was 176 persons per group, 352 in total.

### Randomization

Participants were randomized to either EUC or the psychosocial counselling intervention (PSY) using simple randomization and an online randomization chart on 1:1 basis. After obtaining informed consent from the participant, the research assistants called the study coordinator, who carried out the randomization at a site remote from the trial location and gave the result on the phone. The allocation was thus concealed to participants and research assistants.

### Blinding

Blinding of participants and research counsellors was not possible due to the nature of the intervention. The baseline, 1-month and 6-month assessments were performed by research assistants who had received a 5-day training and were blinded to the allocation status of the participants.

### Statistical analysis

We used descriptive statistics to assess the balance between trial arms at baseline. The primary between-group analysis was carried out using an intention-to-treat approach. Multilevel mixed-effects models were used to account for the correlation among the repeated observations of the same subject over time (Tang & Tu, 2012). Mixed models allow to vary the number of observations within each participant, handling missing data more effectively than other analytical approaches (O'Connell *et al*., 2017; Rahman *et al*., 2016). The likelihood ratio (LR) test was used to compare the multilevel model with a single-level regression. We also used the LR test to examine differences by health post, which did not improve the fit of the model (*p* < 0.00001). Therefore, we only adjusted for the repeated measurements and not for clustering at the health post level.

The primary outcome (response to treatment) was calculated using the following formula: 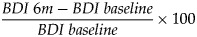. This per cent change was then dichotomized into 1 (50% or more) or 0 (less than 50%). A multilevel mixed-effects logistic regression model was fitted. Time as dummy variables and an interaction term between intervention group and time (as dummy variable) were included as fixed effects and participant as a random effect. Predicted margins were used to obtain predicted proportions and percentage difference between intervention groups and 95% confidence intervals. We used the *melogit* command that integrates fixed and random effects since version 14 of Stata.

Similar analyses were carried out for secondary outcomes using linear mixed-effects models. The model adjusts for baseline values to account for regression to the mean. Differences in mean scores were obtained from equation coefficients (Twisk *et al*., 2018). The standardized mean difference for BDI was calculated by dividing the difference in mean scores by the pooled standard deviation using the formula from da Costa *et al*. (2012). Mean BDI, BAI, and WHODAS at baseline, T1 and T2 were obtained by calculating predicted fitted values which integrate fixed and random effects.

All analyses were carried out with Stata version 14.2 (Stata Corp., 2015). The code is available upon request.

## Results

**Fig. 1.**
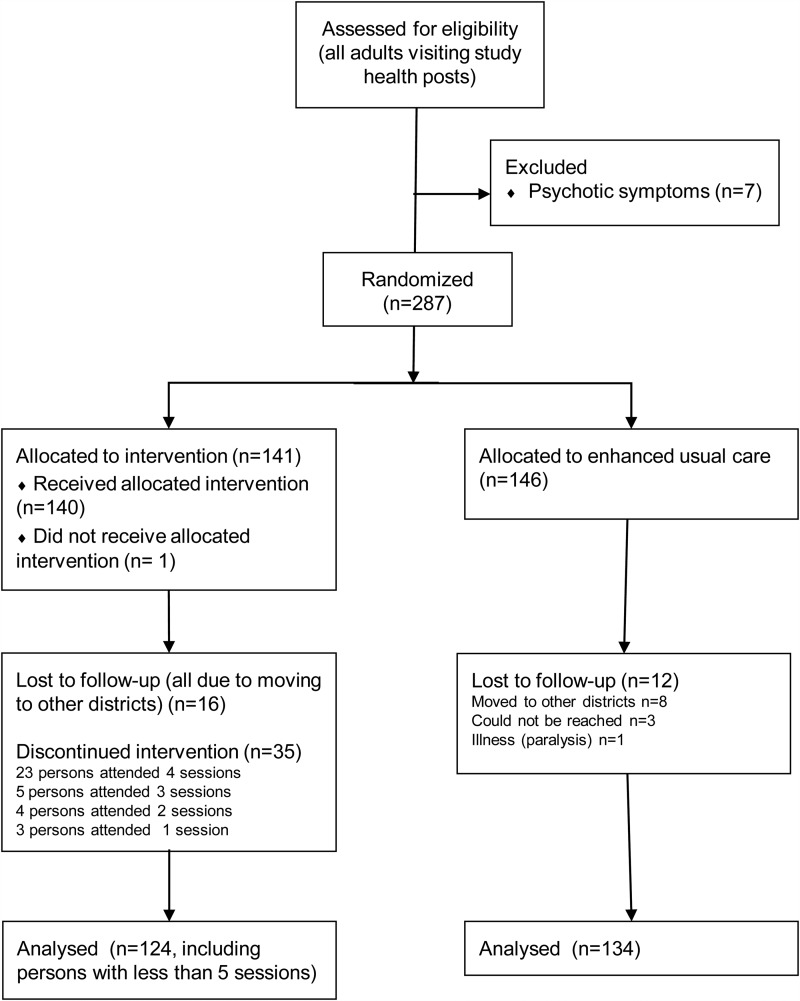
Flow chart of study participation.

A total of 141 participants were randomized to receive psychosocial counselling (PSY) and 146 participants were randomized to EUC (Fig. 1). Data collection began in May 2016 and ended in October 2017. Seven persons were excluded at the screening phase due to psychotic symptoms, and no one for suicidality. No participants had suicidal behaviour during the study, and no participant was excluded during the study. Participants at baseline were predominantly female, with a low level of education, and married with large families (Table 1).

**Table 1.** Baseline characteristics of 286 trial participants in rural Nepal by intervention group

	Psychosocial counseling (PSY) *n* = 141	Enhanced usual care (EUC) *n* = 146
Women	127 (90.7%)	135 (92.5%)
Age (years)	29.1 (10.6)	33.8 (11.7)
Illiteracy	17 (12.1%)	27 (18.5%)
Education		
Informal	31 (25.4%)	57 (47.1%)
Primary	17 (14%)	19 (15.7%)
Lower secondary	37 (30.3%)	15 (12.4%)
High school or above	37 (30.3%)	37 (27.7%)
Ethnicity		
Brahmin	28 (33.3%)	28 (27.2%)
Tharu	48 (57.1%)	58 (56.3%)
Magar	8 (9.5%)	15 (14.6%)
Other	0	2 (2%)
Religion		
Hindu	124 (87.9%)	132 (91%)
Muslim	2 (1.4%)	1 (0.7%)
Christian	15 (10.7%)	12 (8.3%)
Marital status		
Unmarried	21 (14.9%)	11 (7.5%)
Married	108 (77.3%)	115 (78.8%)
Widow, separated or divorced	11 (7.8%)	20 (13.7%)
Family members (mean)	5.8 (2.6)	6.3 (3.3)
BDI score mean	25 (8.9)	22 (8.6)
BAI score mean	21.9 (9.8)	19.4 (9.1)
WHODAS score mean	20 (9.4)	18 (10.4)

Follow-up at T2 was achieved for 87.9% of the intervention group and for 91.8% in the control group. Total attrition was 10.1%. Reasons for lost to follow-up are described in Fig. 1.

Table 2 shows the response to treatment by intervention group. In PSY, 101 participants out of 124 (80.5%) had a response (>50% reduction in BDI score), compared with 57 participants out of 134 (41.1%) in EUC. The percentage difference of PSY *v.* EUC was 39.4% (95% CI 28.4–50.4).

**Table 2.** Response to treatment and percentage difference at 1 and 6 months by intervention group

	1 month		6 month	
	Response to treatment	Percentage difference PSY *v*. EUC	Response to treatment	Percentage difference PSY *v*. EUC
Intervention	78 (65.0%)	44.8% (34.6–54.9)	101 (80.5%)	39.4% (28.4–50.4)
Control	28 (20.2%)		57 (41.1%)	

The mean differences adjusted for baseline values in BDI, BAI, and WHODAS between T0 and T2 are shown in Table 3. At T2, the scores were 6.6 (95% CI 5.5–7.6) in PSY and 14.0 (95% CI 12.7–15.3) in EUC. Compared to EUC, at T2 the intervention group had a lower BDI score (mean difference −7.43 95% CI −9.71 to −5.14), a lower BAI score (mean difference −5.42, 95% CI −7.59 to −3.27), and the lower WHODAS score (mean difference −5.04, 95% CI −7.33 to −2.74). The standardized mean difference for BDI score was 0.72.

**Table 3.** Mean BDI, BAI, and WHODAS and difference mean scores at baseline and 6 months

	Mean score (95% CI)		
	Psychological counselling	Enhanced usual care	Difference mean scores at 1 and 6 months
BDI			
Baseline	25.0 (24.1–26.0)	22.0 (20.8–23.3)	
1 month	10.6 (9.5–11.6)	18.1 (16.8–19.4)	−7.48 (−9.76 to −5.21)
6 months	6.6 (5.5–7.6)	14.0 (12.7–15.3)	−7.43 (−9.71 to −5.14)
BAI			
Baseline	21.9 (21–22.9)	19.4 (18.2–20.5)	
1 month	9.3 (8.3–10.3)	15.4 (14.3–16.6)	−5.87 (−8.02 to −3.72)
6 months	5 (4–6)	10.8 (9.6–11.9)	−5.42 (−7.59 to −3.27)
WHODAS			
Baseline	20 (18.9–21.1)	18 (16.6–19.4)	
1 month	9.1 (8–10.3)	15.1 (13.7–16.5)	−5.67 (−7.95 to −3.38)
6 months	5.7 (4.5–6.8)	10.9 (9.5–12.3)	−5.04 (−7.33 to −2.74)

**Fig. 2.**
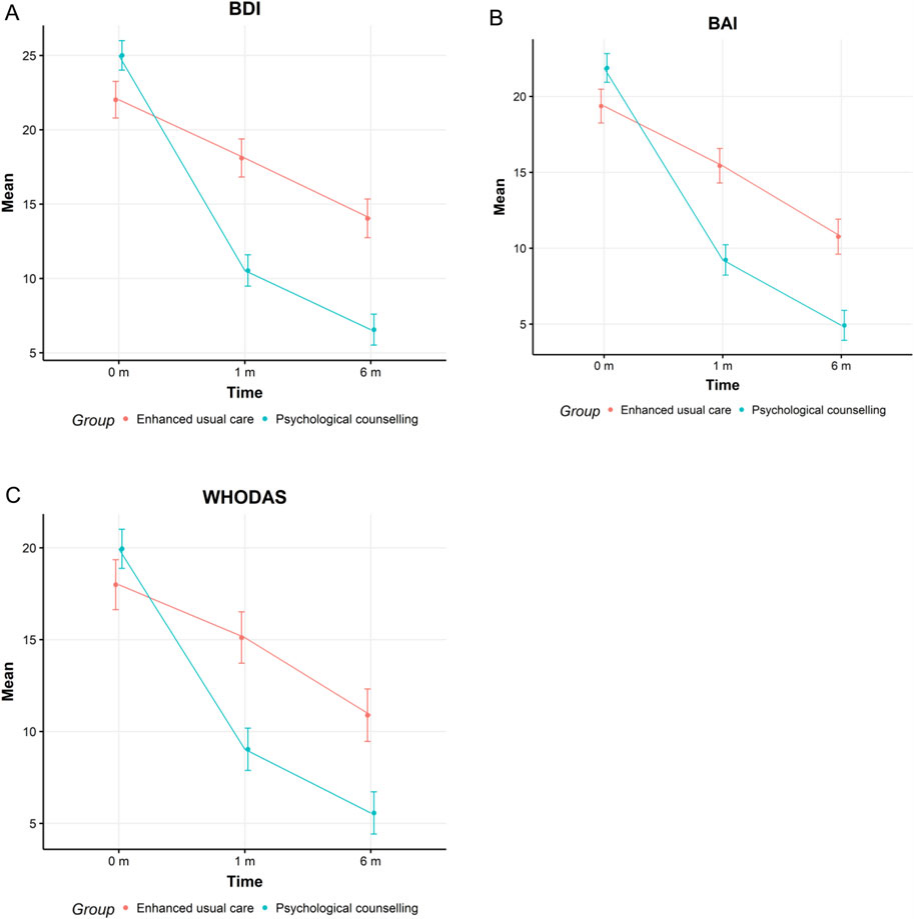
The mean Beck Depression Inventory (A), Beck Anxiety Inventory (B) and WHO Disability Assessment Schedule (C) scores in intervention (blue) and control (red) groups at 0, 1 and 6 months.

The corresponding figures for BAI and WHODAS are presented in Fig. 2*a*–*c*.

### Adherence to the intervention

The trial was planned to encompass five sessions, however, for more severe cases, one–two additional appointments were scheduled if requested by the participants. Altogether 104 participants (74.4%) received a minimum of five sessions (61 participants received six sessions and nine participants seven sessions). The mean number of sessions received was 5.2. The duration of treatment sessions was 35–60 min, which in some cases differed somewhat from the planned 45 min due to study participants' preferences.

## Discussion

This randomized controlled trial aimed to assess the effectiveness of non-medical health worker-led psychosocial counselling in a rural setting in Nepal. The intervention was remarkably effective: 81% of persons who received the intervention had a response (minimum 50% reduction in BDI score), compared with 41% of persons in the control group. Also, secondary outcomes, the BAI and the WHODAS, showed a substantial reduction that was larger in the intervention than in the control group.

### Comparison with previous studies

The magnitude of the effect of our intervention is in line with a meta-analysis of 27 trials of lay psychosocial treatments that found a standardized mean difference of 0.49 (Singla *et al*., 2017). The review identified different elements of lay psychological treatments of which the interpersonal, emotional, and nonspecific engagement showed the strongest association with intervention effectiveness (Singla *et al*., 2017). Our intervention emphasized the engagement between the client and the counsellor and interpersonal elements, which are culturally relevant content. Approaches such as breathing exercises and relaxation are widely used in Nepal to relieve stress (Pramanik *et al*., 2010; Naik *et al*., 2018), and we consider them a cultural adaptation in this counselling intervention. Surprisingly, relaxation was one of the least often used methods in other similar trials (Singla *et al*., 2017).

The structure and delivery method of our intervention were similar to the majority of other studies reviewed by Singla *et al*. (2017). In most studies, the psychological treatment was delivered by community health workers, followed by peers or lay persons, as in our case.

Varying definitions of lay health workers exist: for example, in the successful trial of Chibanda *et al*. (2011), health workers received a 9-day training in counselling skills and problem-solving therapy. In terms of training length, this is close to the training provided in this trial to the health workers providing EUC (5 days of training and 3 days of refresher training), although the content was not focused on counselling. In the literature, the duration of the training for nonspecialist providers has varied from 3 h to 2 months (Singla *et al*., 2017). Compared to this, the 6-month training of the counsellors in this study is significantly longer and could have contributed to our high response rate and low drop-out rate. Therefore, the generalization of these results to settings with less trained or experienced lay counsellors is not straightforward. On the other hand, the mean duration of the treatments in the review was 10 weeks with a mean of 9.6 sessions, whereas the intervention provided in this study lasted 4 weeks (5.2 sessions).

### Public health implications

Currently, the coverage of basic psychosocial treatment for depression in Nepal is estimated at 1% (Chisholm *et al*., 2016*a*). To increase the coverage to 30%, along with treatment coverage of other more severe psychiatric problems and including both psychosocial treatment and medications, is estimated to cost 0.56 USD per capita (Chisholm *et al*., 2016*a*). Only a fraction of these costs would be due to psychosocial treatment. This figure, even though small, is 14-fold to the current expenditure of 0.04 USD per capita. Considering this lack of resources and the extremely low numbers of formally trained mental health professionals, using lay health workers as service providers seems like the only possible option to achieve an increase in the coverage of psychosocial treatments in Nepal in the near future.

In this study, the lay counsellors received a 6-month training, which is longer than in most task shifting studies (Singla *et al*., 2017). The long duration of the training could be seen as a limitation for scaling up the intervention. However, the tuition fee of training one person as a counsellor is approximately 1000 USD, and after the initial investment, the trained lay counsellors are able to provide effective treatment with relatively little support, as seen in this study. Additionally, in the development project that this research was related to, counsellors with the same training and experience had other responsibilities in addition to counselling: they had a supervisory and supporting role with the government health workers and female health volunteers providing basic psychosocial support, and run outreach workshops at communities and schools. Therefore, well-trained psychosocial counsellors can potentially have a key role in mental health care at the local level beyond the provision of counselling services.

Recently, a systematic review confirmed that psychotherapy may substantially reduce psychological symptoms in areas that have suffered from disasters and conflict (Purgato *et al*., 2018). The psychotherapies in the reviewed trials were mostly provided by mental health professionals. This study demonstrates that also non-medical health worker-led counselling can be useful in a region that has suffered a recent violent conflict and several natural disasters.

The reason for using lay health workers to provide psychosocial therapies is simple: there will never be enough mental health professionals in any country to deliver psychological treatments for all that could benefit from them. In addition to specific techniques, nonspecific qualities such as the ability to form an empathic relationship should also be assessed when recruiting lay therapists (Singla *et al*., 2017). Strong support and supervision is generally considered a requirement for effective lay person delivered mental health care (Barnett *et al*., 2018) and could be a challenge for scaling up these interventions (Singla *et al*., 2017). However, in this study, supervision was provided flexibly as needed by other, more experienced counsellors, a field coordinator, and a medical doctor by phone, and did not require an excessive amount of resources. Based on this experience, it appears that a structured, intensive supervision system may not be mandatory for successful lay counselling, as long as some support is available when needed. Nevertheless, this approach to supervision requires adequately trained and experienced lay counsellors.

Thus, compared with earlier literature, this study demonstrates that the requirements for supervision and length of the intervention may be less than previously thought if it is delivered by adequately trained lay counsellors. Moreover, adding indigenous elements, such as relaxation and breathing exercises, may be beneficial for effectiveness.

Additionally, it should be noted that the study was carried out in the context of a development cooperation project by two NGOs. It is our firm belief that development projects should apply evidence-based interventions, and if possible, should try to incorporate elements to assess their effectiveness with rigorous scientific methods.

This study was part of a development cooperation project that created structures to support the provision of mental health services in the district in the future: with support of local authorities, district and village level mental health committee were founded, in addition to training of health workers and sensitization of the population through various outreach activities. This broader approach not only aided in the recruitment of study participants but also helped to sustain the efforts. After 6 years of continuous work in the district, mental health is now incorporated into local primary health care, and counsellors were trained and hired by local health authorities to work at the health posts. After sufficient training, primary health workers have been content with their role of providing basic psychosocial support and referring to counsellors if needed, while counsellors have also had a supervisory role, and mental health care is provided as an integral part of primary health care.

### Limitations

Some limitations of the study should be noted. First, the study population included few men, and therefore the effectiveness of the intervention could not be assessed, and the results of the study cannot be generalized to men. Second, the follow-up did not extend beyond 6 months, and the long-term impact of the intervention is unknown. Third, we were not able to collect detailed information on other treatments received, such as the use of antidepressant or other medication or possible visits to other health-related local resources such as traditional healers. However, the study counsellors did not prescribe medications, and therefore any differences between the two groups are not likely to be due to the use of psychotropic medication. Fourth, even though randomization procedures were adhered to, there were some differences between the two study groups in relation to education. Fifth, the sample size calculation was based on the initial idea of using continuous BDI as the primary outcome, but later, a categorical outcome of 50% reduction in BDI was chosen. However, the sample size was sufficient for the categorical outcome and the study was adequately powered to detect the impact of the intervention. Finally, the impact of caste was not assessed specifically in this trial. Over half of the participants were Tharus, an indigenous underprivileged population residing in the Southern Himalaya Region. Low castes (Dalit/Nepali) have been found to have a greater prevalence of depression and anxiety when compared with high castes. In Nepal, caste is an important mediator of mental health (Kohrt *et al*., 2009), and its impact should be assessed in further trials.

## Conclusions

Upscaling the availability of psychosocial treatments is a global challenge. In the past, the global mental health movement has been criticized for being excessively focused on the biomedical treatments for mental disorders, but recently, efforts have been made to include more psychosocial components into basic care packages (Hanlon *et al*., 2016). This randomized controlled trial showed the effectiveness of non-medical led psychosocial counselling in reducing depressive and anxiety symptoms and disability in a challenging post-conflict low-income setting. Further trials in Nepal should be scaled-up nationwide to provide more generalizable findings. Low and middle-income countries should consider including lay psychosocial counselling as an integral part of mental health systems.

## Supplementary material

For supplementary material accompanying this paper visit http://dx.doi.org/10.1017/gmh.2019.15. click here to view supplementary material
