# Effectiveness of non-medical health worker-led counselling on psychological distress: a randomized controlled trial in rural Nepal – ADDENDUM

**DOI:** 10.1017/gmh.2019.18

**Published:** 2019-08-23

**Authors:** N. Markkula, V. Lehti, P. Adhikari, S. Peña, J. Heliste, E. Mikkonen, M. Rautanen, E. Salama, B. Guragain

Keywords: Common mental disorders, lay counselling, Nepal, randomized controlled trial, task shifting, addendum

The authors of Markkula, et al (2019), would like to acknowledge the help and support given throughout the process by Dr Bhogendra Sharma, President of Centre for Victims of Torture. They also wish to thank Dr Ram Prasad Sapkota, Dr Elina Hemminki, Dr Jaana Suvisaari and Dr Ulla Ashorn for their valuable inputs in earlier phases of the study.
