# Supplementary material for: Effectiveness of non-medical health worker-led counselling on psychological distress: a randomized controlled trial in rural Nepal
Source: Glob Ment Health (Camb). 2019 Jul 16;6:e15. doi: 10.1017/gmh.2019.15 (PMC6669965; doi:10.1017/gmh.2019.15)
Supplement: Supplementary file 1 [file S2054425119000153sup001.zip › S2054425119000153sup001/CONSORT Extension for Abstracts Checklist _SPF.doc]

**Items to include when reporting a randomized trial in a journal or conference abstract**

| **Item** | **Description** | **Reported on line number** |
| --- | --- | --- |
| Title | Identification of the study as randomized | YES |
| Authors * | Contact details for the corresponding author |  |
| Trial design | Description of the trial design (e.g. parallel, cluster, non-inferiority) | YES |
| Methods |  |  |
| Participants | Eligibility criteria for participants and the settings where the data were collected | YES |
| Interventions | Interventions intended for each group | YES |
| Objective | Specific objective or hypothesis | YES |
| Outcome | Clearly defined primary outcome for this report | YES |
| Randomization | How participants were allocated to interventions | YES |
| Blinding (masking) | Whether or not participants, care givers, and those assessing the outcomes were blinded to group assignment |  |
| Results |  |  |
| Numbers randomized | Number of participants randomized to each group | YES |
| Recruitment | Trial status | YES |
| Numbers analysed | Number of participants analysed in each group | YES |
| Outcome | For the primary outcome, a result for each group and the estimated effect size and its precision | YES |
| Harms | Important adverse events or side effects | NO |
| Conclusions | General interpretation of the results | YES |
| Trial registration | Registration number and name of trial register | YES |
| Funding | Source of funding | YES |

**this item is specific to conference abstracts*
